# Sharing fishers´ ethnoecological knowledge of the European pilchard (*Sardina pilchardus*) in the westernmost fishing community in Europe

**DOI:** 10.1186/s13002-017-0181-8

**Published:** 2017-09-14

**Authors:** Heitor de Oliveira Braga, Miguel Ângelo Pardal, Ulisses Miranda Azeiteiro

**Affiliations:** 1 Centre for Functional Ecology - CFE, Department of Life Sciences, University of Coimbra, Calçada Martins de Freitas, 3000-456 Coimbra, Portugal; 2 CAPES Foundation, Ministry of Education of Brazil, Caixa Postal 250, Brasilia, DF 70040-020 Brazil; 3 Department of Biology & CESAM - Centre for Environmental and Marine Studies, University of Aveiro, 3810-19 Aveiro, Portugal

**Keywords:** Ethnoecology, Folk knowledge, Fishermen, European pilchard, Participatory management

## Abstract

**Background:**

With the present difficulties in the conservation of sardines in the North Atlantic, it is important to investigate the local ecological knowledge (LEK) of fishermen about the biology and ecology of these fish. The ethnoecological data of European pilchard provided by local fishermen can be of importance for the management and conservation of this fishery resource. Thus, the present study recorded the ethnoecological knowledge of *S. pilchardus* in the traditional fishing community of Peniche, Portugal.

**Methods:**

This study was based on 87 semi-structured interviews conducted randomly from June to September 2016 in Peniche. The interview script contained two main points: Profile of fishermen and LEK on European pilchard. The ethnoecological data of sardines were compared with the scientific literature following an emic-etic approach. Data collected also were also analysed following the union model of the different individual competences and carefully explored to guarantee the objectivity of the study.

**Results:**

The profile of the fishermen was investigated and measured. Respondents provided detailed informal data on the taxonomy, habitat, behaviour, migration, development, spawning and fat accumulation season of sardines that showed agreements with the biological data already published on the species. The main uses of sardines by fishermen, as well as beliefs and food taboos have also been mentioned by the local community.

**Conclusions:**

The generated ethnoecological data can be used to improve the management of this fishery resource through an adaptive framework among the actors involved, in addition to providing data that can be tested in further ecological studies. Therefore, this local knowledge may have the capacity to contribute to more effective conservation actions for sardines in Portugal.

**Electronic supplementary material:**

The online version of this article (10.1186/s13002-017-0181-8) contains supplementary material, which is available to authorized users.

## Background

Human populations have forced marine coastal ecosystems to differ from their historical states, which were characterized by diversified and productive communities [1]. One of the biggest human impacts has been overfishing, which has progressively reduced stocks, geographically expanded its range and disguises itself through new and improved technologies [2].

In marine ecosystems, pelagic fish are recognized as abundant in productive fishing areas, both on a large scale and on a small scale [3], and are characterized by a history of large fluctuations in their populations, both due to overfishing as well as environmental factors [4]. Within this group of fish, we have small pelagic species, such as sardines and anchovies, which are abundant in several productive regions of the ocean and are found mainly in areas of coastal and oceanic upwelling [5]. These clupeoid fishes are recognized mainly as having a low trophic level in the food web, early reproduction age and rapid growth, all of which make them more vulnerable to different environmental factors and climate change [5].

In the central, eastern, and northeastern Atlantic, the European pilchard *Sardina pilchardus* (Walbaum, 1792) stands out among the pelagic fish for fisheries [6]. At the moment in Iberian waters, this species exhibits low biomass stocks at age 1 and a decrease in the stock of old fish and low recruitment rates [7]. In Portugal, European pilchard are one of the most important species to fishing fleets using purse seines and are recognized for their socio-economic values and traditional uses among the Portuguese [3].

With all these processes occurring, it is important to understand both the perceptions of local fishery managers and users of local resources and to provide strategies for avoiding conflicting shared perceptions among the stakeholders involved in fisheries management [8]. In small-scale fisheries, for example, local fishermen are in many cases disadvantaged in relation to the actors belonging to large-scale fisheries due to their marginal political power, lack of infrastructure and their typical remoteness [9].

However, it is known that support from the general public for the management of natural resources is fundamental for long-term sustainability [10]. Discussions should be initiated with these local communities as a way of transferring responsibility and regulatory power over available environmental resources [11].

The local ecological knowledge (LEK) in this context serves as an effective tool for monitoring and assisting in the planning of depleted resources, for the conservation of biodiversity [12], and for conducting more reasonable and culturally sensitive research and management plans [13]. This knowledge can be understood as the lay or experiential knowledge of an individual about the environment based on daily observations, practical experiences in nature and learned scientific knowledge [14].

To better understand recent history of artisanal fishing and the deterioration of the standard of living of the dependents of this resource [15], we can employ ethnoecology, which according to Marques (2001) can be understood as the scientific study of traditional ecological knowledge (knowledge, behaviour, feelings, and beliefs that influence all interactions between humans and the ecosystem) [16]. More specifically within ethnozoology, we have ethnoichthyology [17], which aims to report the knowledge that fishermen have about fish biology and ecology [18], and the understanding of interactions between humans and ichthyological resources encompassing the cognitive and behavioural aspects supported by conservation [19].

From this perspective, extracting ethnoecological data about European pilchard from the fishing community of Peniche, as well as the knowledge passed from generation to generation by the more experienced fishermen, can be important strategies for the conservation of this fishing resource. This type of ethnoecological survey emphasizes the cultural knowledge of fishermen, favours their dialogue with environmental managers and researchers, and contributes to the improvement of participatory management of natural resources by increasing the acceptance of management rules [20].

Thus, the aim of this study was to record the ethnoecological knowledge of the fishing village of Peniche, Portugal, about the ecology and biology of *S. pilchardus. T*he fishermen’s profiles and the likely human uses, beliefs and taboos related to European pilchard (also known as the Atlantic sardine, European sardine, or sardine) were also explored. The ethnoecological data provided by the fishermen who are in agreement with the published biological data were interpreted and discussed in the present study.

## Methods

### Study area

**Fig. 1:**
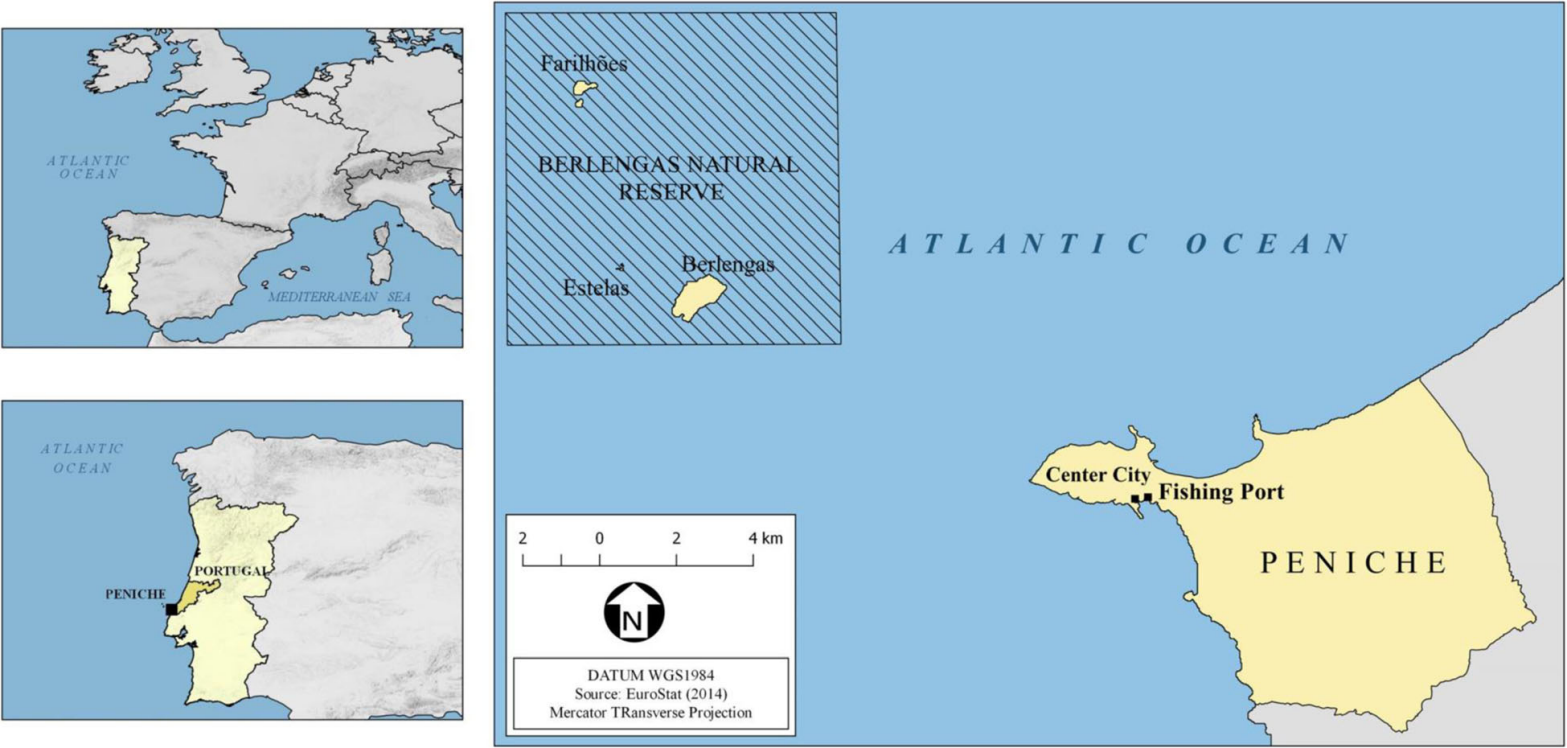
Map of the study area, highlighting the fishing port and the city center of Peniche where the interviews were conducted in Portugal. Credits: B Zucherato

This study was based on interviews with artisanal fishermen from the fishing community of Peniche, on the coast of the western sub-region of Portugal (39° 21′ 32″ N, 9° 22′ 40″ W; Fig. 1). This city has 27,628 inhabitants with an area of approximately 77,55 km2 [7]. The climate is temperate with rainy winters and dry and somewhat hot summers (Köppen type Csb) [7].

One of the world’s first Portuguese protected areas [21] is located approximately 5.7 miles from Peniche (Cape Carvoeiro) in the Atlantic Ocean [22]. Formed by an archipelago of islands (Berlenga Grande, Estela and Farilhões), this marine protected area is located in the transition zone between the Mediterranean and European sub-regions, specifically at the top of the escarpment of the Nazaré Canyon [22]. The Berlengas Marine Natural Reserve (MNR) is renowned for its great marine biological diversity, archaeological features, insular ecosystem specificities and is importance in the life cycle of the marine avifauna [23].

### Fishing community

The fishing port of Peniche is recognized as one of the main ports of the country [24] according to fishing indicators for the average value of fish unloaded in this area [7, 25]. The economic and social development of the city is directly linked to the fishing activity of this port, which is one of the busiest in Portugal [26].

This fishing community is considered a local symbol with remarkable prestige throughout the municipality [25]. In the maritime captaincy of Peniche, there are approximately 1105 registered fishermen, with 996 conducting marine fishing [7]. Polyvalent and seine fishing are predominant in this area, with sardines being one of the three main target species for fishing according to the data on the nominal catch landed in Portugal [7].

### Fishermen’s Interviews

Semi-structured interviews [27, 28] were conducted from June to September 2016 to obtain data on LEK about the European pilchard. The state-owned company Docapesca - Portos e Lotas granted permission for administering the interviews to the fishermen of Porto de Peniche. Fishermen were interviewed over successive visits in the fishing warehouses of the Port of Peniche and at the main meeting points of the fishing community. Brook and McLachlan (2008) sees this type of involvement with the community as indispensable [29]. The objectives of the work and the statement of informed consent to participate in the research were provided to the fishermen through the Statement of Informed Consent (IC) [see Additional file 1].

The interviews were mainly conducted through manual transcription and occasionally with a digital audio recorder. The interviews with fishermen were conducted randomly - always before or after the arrivals and departures of the fishing teams and when they were doing net and fishing gear repairs. The interview script [see Additional file 2] was structured in 2 parts: Profile of fisherman (age, schooling, fishing time, time of residence in Peniche, income source, type and length of boat, fishing time at sea and time to catch sardines) and LEK of European pilchard (folk taxonomy, habitat, behaviour and migration, development of sardines, spawning, fat accumulation season and uses, beliefs and food taboos). The educational profile of the interviewees followed the Portuguese educational classification: A (1st Cycle: 1–4 years of study), B (2nd Cycle: 5–6 years of study), C (3rd Cycle: 7–9 years of study) and D: (Secondary Education: 10–12 years of study).

### Data analyses

A respect for the stakeholders and communities, the clarification of data collection objectives, the interactive approach and the recognition of information limitations were used as a basis for analysing the data acquired [30]. All the information provided through the surveys was analysed following the union model of the different individual competences [31]. The LEK about sardine was analysed through an emic-etic approach [32], and the data generated by the community were compared with the scientific literature [16]. The wealth of information and depth of perceptions in the data collected were analysed through careful coding and cross-checking to ensure the objectivity of the study [27]. Species nomenclature data were analysed following the Food and Agriculture Organization of the United Nations (FAO) [33], the International Union for Conservation of Nature (IUCN) [34], and Fish Base [35]. The data obtained in the interviews were stored and standardized in EXCEL and analysed (descriptive statistics) in the R Project for Statistical Computing version 3.3.2 [36].

## Results

### Descriptive statistics of fishermen’s profiles

**Fig. 2:**
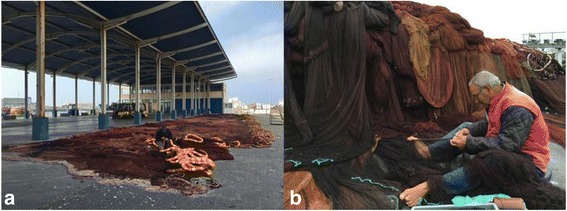
**a** Main area of the fishing port of Peniche, Portugal where the interviews with the fishermen were carried out. **b** A fisherman doing maintenance of purse seine nets. Credits: HO Braga. (Images published under previous consent of the participants)

A total of 87 interviews were conducted in the fishing community of Peniche. The interview sites were predominantly in the Port of Peniche (Fig. 2) (*N* = 71) followed by the city centre (*N* = 16). The average age of the respondents was 58.25 years, with a minimum of 25 years and a maximum of 76 years. More than half of the interviewees (*N* = 59) were born in Peniche and are active (*N* = 49) in the fishing currently. According to the Portuguese educational classification, 58 individuals belonged to the 1st Cycle, 19 to the 2nd Cycle, 5 to the 3rd Cycle and 5 to Secondary Education. There were no illiterates or individuals with higher education in the sample. The fishing experience varied from 3 to 60 years, but fishing experience average was 39.08 years. The average monthly income source related to fishing, including retirees, is 810.5 Euros, ranging from 208.0 to 3000.0 Euros. A total of 50 fishermen interviewed supplement their income with income from other activities. Of these fishermen, 31 informants do so through work related to fishing (maintenance of fishing nets) and the rest in other autonomous activities.

The main types of boats mentioned by the fishermen were: trawlers, artisanal fishing boats, trawl nets, coastal boats, and sports boats. Trawlers were further sub-classified by fishermen into trawlers (larger boats) and “rapa” (smaller boats). Boats were measured 93 times during the interviews. Of these, the boats mentioned were as follows: trawlers (35), artisanal fishing boats (38), “rapa” (14), trawl nets (3), coastal boats (2) and sport boats (1). Six Fishermen have said they have fished in more than one type of boat, those being 3 in both artisanal fishing boats and trawlers and 3 in artisanal fishing boats and trawl nets. Only 17 of the fishermen interviewed are boat owners.

The fishermen interviewed were questioned about their preferred schedule of sardine capture during the fishing campaigns*.* A cycle called "It is six in the morning and six in the afternoon" was the most cited by fishermen (*N* = 27). Other respondents mentioned both sunrise and sunset (*N* = 17), day and night (*N* = 16), preferably day (N = 16), preferably night (*N* = 9), and only at sunset (N = 2).

### Local ecological knowledge of sardines

#### Folk taxonomy

In Peniche, besides the name sardine, the artisanal fishermen attributed popular names to small sardines. A total of 82 fishermen attributed the name “petinga” or “esquilha” to juvenile sardines. One fishermen mentioned the name “real” sardine and another fishermen “sueste” fish. In this community, only two fishermen mentioned the scientific name (Linnaean).

#### Habitat, behaviour and migration

When questioned about the preferential habitat of sardines, 40 of the fishermen indicated coasts and high sea, 39 only on the coast, 6 more often on the coast, one only on the high sea and one did not answer this question. The informants highlighted some specific habitats (rocky seabeds, areas where the river empties into the sea carrying food, clean seabeds, more temperate waters, and sandy seabed to escape from common dolphin (*Delphinus delphis* Linnaeus, 1758) attacks.

In relation to the most common depth of the sardine in the sea, 32 informants indicate a forage interval of 0–50 m, 40 between 0 and 100 m and 11 between 0 and 200 m. Two fishermen did not know how to answer this question and two others just said they were deep-sea fish.

The locomotion of sardines in the sea, according to all fishermen, is carried out in shoals. Some fishermen (*N* = 15) specified that the schools are enormous. Two other fishermen referred to a phenomenon that makes sardines stick together. One informant said that sardines usually come together to protect themselves from common dolphins, and the other informant said that common dolphins make them stay together to feed.

The following types and patterns of sardine migration have been mentioned by fishermen in the fishing community of Peniche: the migration comes from the South (13 times), comes from the North (18 times), comes from the South and North (32 times), occurs with the tides (3 times), occurs according to the seasons of the year - summer and winter (15 times) and migrant/pelagic/moving fish (14 times). Only 7 respondents did not respond to this part of the interview.

Local fishermen also mentioned the probable areas of sardine displacement along the Portuguese Coast that passes through Peniche (Table 1). The areas most cited by respondents were Figueira da Foz (27 times), Algarve (25 times), Nazaré (19 times), Ericeira (15 times), Sesimbra (12 times) and Sines (11 times). Other areas of Portugal, such as Aveiro, Setúbal and Portimão (8 times), Cape Roca (7 times), São Pedro de Moel and Matosinhos (4 times), Viana de Castelo, Póvoa de Varzim, Olhão and Santa Cruz (3 times) and Cascaiz and Leixões (2 times). Foz do Minho Beach, Vila do Conde, Caparica Coast, Porto Beach, Mira Beach, Tocha Beach, Sagres and Lisbon were mentioned only once in the interviews.

**Table 1:** Probable areas of sardines displacement along the Portuguese Coast according to the fishermen of Peniche

Fishing spots in Portugal	Number of times cited by fishermen
Figueira da Foz	27
Algarve	25
Nazaré	19
Ericeira	15
Sesimbra	12
Sines	11
Aveiro, Setúbal and Portimão	8
Cape Roca	7
São Pedro de Moel and Matosinhos	4
Viana de Castelo, Póvoa de Varzim, Olhão and Santa Cruz	3
Cascaiz and Leixões	2
Foz do Minho Beach, Vila do Conde, Caparica Coast, Porto Beach, Mira Beach, Tocha Beach, Sagres and Lisbon	1

### Development of sardines

In the development section, the fisherman was asked about the time of sardine growth. According to 57 of respondents, sardines showed rapid growth, 16 said they were slow growing, 3 indicated intermediate growth (neither slow nor fast) and 11 did not know how to answer this question. The vast majority of respondents (*N* = 85) said that the sardine exhibit only the roe phase during their development. One fisherman mentioned both the larval stage and the roe phase, and another fisherman did not know how to answer this question.

Only 19 of respondents said that sardine mature after 1 year of age, and 24 did not know how to respond. The rest of the interviewees (*N* = 44) said that the sardine is able to reproduce within a range of 3–7 months old.

### Spawning and fat accumulation season

The answers about the spawning time of sardines varied among fishermen. Most informants cited the spawning months as the answer to this question. The spawning time ranged from one to 8 months (72 citations). There was 1 response for 8 months, 2 for 4 months, 3 for 6 months and 3 for 7 months, 7 responses for 5 months, 16 for 4 months, 17 for 3 months and 20 for 2 months. The rest of the respondents (*N* = 3) did not report any months.

According to respondents, spawning occurs mainly in the months of January and February (20 times). December was quoted 19 times, November and March 15 times and October 12 times. The months of April (9 times), May and September (6 times), June (5 times), July and August (4 times) were the least mentioned by fishermen in Peniche (Table 2).

**Table 2:** The sardines spawning period according to the fishermen interviewed

Sardines spawning period	Number of times cited by fishermen
*Months*	
January and February	20
December	19
November and March	15
October	12
April	9
May and September	6
June	5
July and August	4
*Seasons*	
Winter	12
Summer	8
*Time per year*	
2 times	4
2–3 times	4
3 times	3
3–4 times	1

Other fishermen still specified the occurrence of spawning seasons. Along these lines, there were citations only for winter (*N* = 12) and summer (*N* = 8). There were also fishermen who reported the number of times (2× a year = 4 citations, 2-3× = 4 citations, 3× = 3 citations and 3-4× = 1 citation) that they spawn each year (Table 2).

The fishermen interviewed in Peniche provided some information from this part of the questionnaire below:

1. *“The water becomes creamy and milky when the sardine spawns”.*
2. *"From 100% of the spawn, 90% live and 10% die".*
3. *“The sardine buries itself in the sand to spawn and escape predators”.*
4. *“The sardine goes to the rocks to scratch its belly when it is pregnant”.*
5. *“Outside the summer, the sardines are thin and run away”.*
6. *“The sardine passes its belly through the sand to spawn and leaves the eggs for the sea to take later”.*

The informants mentioned the months of the years when the sardines accumulate (April to December). The months of June (56 times), July (75 times), August (68 times), September (47 times) and October (34 times) are the most remembered by the fishermen. (Table 3). Some local sayings were recorded in the interview as:

1. "*In July, the sardine already drips on bread*".
2. *"The sardine grows earlier in the Algarve (Portugal) because of the warm waters".*
3. *“The more rain, the more the sardine gets fat”.*
4. *"In June and July, they are fatter, just like the Christmas sardine".*

**Table 3:** The period of fat accumulation of sardines according to the respondents

Sardines accumulation season	Number of times cited by fishermen
*Months*	
June	56
July	75
August	68
September	47
October	34

### Trophic ecology: Predators and prey

The LEK of the fishermen of the Port of Peniche showed important aspects of the sardine food chain, indicating the main predators and prey according to fishing experiences along the Portuguese coast. The main predators according to the fishermen (Table 4) are dolphins (*atuninha* or *toninha*), sharks and whales (generally), conger eel (*safio*) and yellowfin tuna (*atum-albacora*).

**Table 4:** The correspondence between the Portuguese folk names of the *S. pilchardus* predators and the scientific classification (Linnaean)

Folk taxonomy	Scientific names (Linnaean)
Atuninha or toninha	*Delphinus delphis* Linnaeus, 1758
Sharkes	Generally
Whales	Generally
Safio	*Conger conger* (Linnaeus, 1758)
Albacora	*Thunnus albacares* (Bonnaterre, 1788)
Robalo	*Dicentrarchus labrax* (Linnaeus, 1758)
Cherne	*Polyprion* *americanus* (Bloch & Schneider, 1801)
Pargo	*Pagrus pagrus* (Linnaeus, 1758)
Cavala	*Scomber japonicus* Houttuyn, 1782
Espada-preto	*Aphanopus carbo* Lowe, 1839
Polvo-comum	*Octopus vulgaris* Cuvier, 1797
Goraz	*Pagellus bogaraveo* (Brünnich, 1768)
Pescada-branca	*Merluccius merluccius* (Linneaus, 1758)
Abrótea	*Phycis* *phycis* (Linnaeus, 1766)
Sarrajão or serrajão	*Sarda sarda* (Bloch, 1793)
Raias	*Raja* spp*.*
Cantarilho	*Helicolenus dactylopterus* (Delaroche, 1809)
Tamboril branco	*Lophius* *piscatorius* Linnaeus, 1758
Espadarte	*Xiphias gladius* Linnaeus, 1758
Carapau	*Trachurus trachurus* (Linnaeus, 1758)
Corvina	*Argyrosomus regius* (Asso, 1801)
Sargo-legítimo	*Diplodus sargus* (Linnaeus, 1758)
Sarda	*Scomber scombrus* Linnaeus, 1758
Faneca	*Trisopterus luscus* (Linnaeus, 1758)
Moréia-preta	*Muraena* *augusti* (Kaup, 1856)
Peixe-lua	*Mola mola* (Linnaeus, 1758)
Tainhas	*Mugil* spp.
Gaivota	*Larus michahellis* J. F. Naumann, 1840
Albatroz	Generally
Birds	Generally

The fishermen also mentioned the following: sea bass (*robalo*), wreckfish (*cherne-legítimo*), red porgy (*pargo-legítimo*), chub mackerel (*cavala*), black scabbardfish (*espada-preto*), common octopus (*polvo-comum*), blackspot seabream (*goraz*), European hake (*pescada-branca*), forkbeard (*abrótea*), Atlantic “bonito” (*sarrajão* or *serrajão*), raja rays nei (*raias*), blackbelly rosefish (*cantarilho*), monkfish (*tamboril branco*) and swordfish (*espadarte*).

The following species were cited only once in the interviews: horse mackerel (*carapau*), meagre (*corvina*), white seabream (*sargo-legítimo*), Atlantic mackerel (*sarda*), pouting (*faneca*), black moray eels (*moréia-preta*) and ocean sunfish (*peixe-lua*). The “*tainha*” was also mentioned in the study in a generalized way. Birds were generally cited as sardine predators. The yellow-legged gull (*gaivota-de-patas-amarelas*) and albatross (*albatroz)* and were also mentioned within this group.

**Fig. 3:**
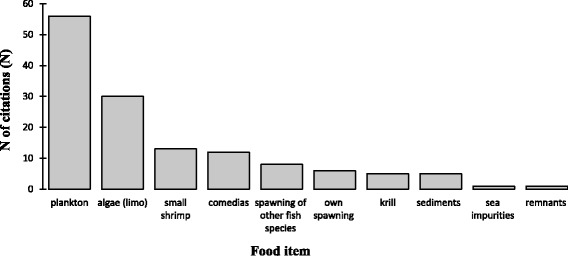
Number of citations of *S. pilchardus* food items by fishermen in the fishing community of Peniche, Portugal

According to the ethnoecological data obtained from the fishing community have a diet (Fig. 3) based on plankton (*N* = 56), algae called “limo” (*N* = 30), small shrimp (*N* = 13), “comedias” or “comedorias” (*N* = 12), the spawn of other fish species (*N* = 8) their own spawn (*N* = 6). The fishermen also said that the sardine feed on krill (N = 5), sediments accumulated after rainfall (N = 5), sea impurities (N = 1), and remnants of other fish species (N = 1). “Comedorias” or “Comedias” in this study was defined by fishermen as a mixture of small fish, small prawns, the roe of other species of fish and sardine roe.

### Human uses, beliefs, and food taboos about European pilchard

The sardine is greatly important to the fishermen interviewed. According to the majority of the interviewees (*N* = 81), sardines have a high economic importance in the region of Peniche. Another 4 fishermen said that the importance of it is average and 2 other respondents have said that the importance is low. The summer (*N* = 21) is cited as the time when the sardine is more important to the population of Peniche. The main uses of this pelagic species are as bait for another species (*N* = 85 fishermen), in the canning industry (*N* = 53), for one’s own food (*N* = 50) and the fish meal industries (*N* = 10). Local commerce (N = 2) and tourism (N = 2) were also identified by Fishermen.

Some informants (*N* = 6) from this community mentioned sardine food taboos. Fish that are restricted were locally termed “raimoso”. A change in the restriction of this fish was observed over time. Twenty-three percent of fishermen said that the sardine was once “raimosa” in the past and 61 % of the fishermen said it was not “raimosa”. Two informants said that the sardine was little “raimosa” and that sick people could not eat it. There were 3 respondents who said that if the joint between the sardine skins is removed, it is no longer a restricted food. The fat found in sardines was recognized as a source of omega 3 by fishermen (*N* = 9), aiding in the medical treatment of people with problems with cholesterol or in the treatment of heart disease patients.

## Discussion

### Folk taxonomy

Local knowledge related to the naming of fish species is an inherent part of fishermen’s trade and can be considered as proof of ability in these communities [37]. In Ericeira, Portugal, the local community also calls the juveniles of this species “petinga” [38]. In the Autonomous Region of the Azores in Portugal, the sardine is also called by the same vernacular name [35]. The designation of sardines as “esquilha” (small fish), “sueste” and “real” are new to the scientific literature.

### Habitat, behaviour and migration

According to the ethnoecological data extracted from the interviews, sardine is a predominantly coastal species and prefers sites near river mouths in the sea. The fishermen reported that sardines are distributed vertically, predominantly between the depths between 0 to 100 m. In the scientific literature, similar information was found indicating that this species is predominantly found in coastal shelf waters [39–43] and prefers areas of great productivity near the mouths of rivers and estuaries [42]. In a study on the modelling of habitat suitability for juveniles of *S. pilchardus*, the results showed that sardines in the growth phase, in search of food and in the spawning process can be closely linked to sites that provide nutrient sources that increase productivity, such as local upwelling or river runoff [44]. Di Natale and collaborators (2011) report in the IUCN Red List of Threatened Species 2011 that European pilchard can usually be found at depths of up to 100 m, reaching a lower depth limit of 180 m [40]. The PECH Committee of the European Parliament for the sardine fishery shows that this species can range down to 150 m [41], and in a study of sardine habitat to the west of Portugal showed that this species has a preference for waters with depths of up to 100 m [43] (Table 5).

**Table 5:** Matrix cognition compared between the fishers´ LEK and the scientific literature on the biology and ecology of European pilchard in Peniche, Portugal

Topics	Fisherman’s citation	Scientific literature
*Habitat*	“Coastal species and prefers sites near river mouths in the sea”. “Depths between 0 to 100 m”.	Coastal shelf waters [39–43]; Areas of great productivity near the mouths of rivers and estuaries [42]; Area of local upwelling or river runoff [44]. Depths of up to 100 m, reaching a lower depth limit of 180 m [40]; Preference for waters with depths of up to 100 m [43].
*Behaviour*	“Migration carried out in shoals”. “Ability to school as a way to ward off predators”.	Migratory behaviour, a high dispersal capacity and schooling behaviour similar to other pelagic fish [45]. Competitors or predators may change the direction or influence the intensity of these migrations in schooling [46].
*Migration*	“Mainly Figueira da Foz and Algarve”. “Póvoa de Varzim, Lisbon...”.	Póvoa de Varzim, Figueira da Foz and Lisbon [49]; Algarve (Southern Portion of Portugal), this pelagic fish is found in greater quantities [49].
*Development*	“Rapid growth”. “Sardine reach sexual maturity at 1 year”; “from 3 to 7 months of age”.	Very fast growth rate [42, 45, 50]. Matures early [52, 53].
*Spawning*	“The main months of spawning are also December, January and February”. “Spawn time can range from one to 8 months”. “In the winter, the sardine spawns more”; “The spawning occurs 2 to 4 times a year”.	October to April [56]; mainly between December and February along the Portuguese coast [57]. Ranging from 3 months per year up to 8 months [43]. Sardines exhibit a prolonged spawning period during the year, with more pronounced spawning mainly in the colder months of the year [43].
*Fat accumulation season*	“June through October”.	Late summer and autumn [58]. Late spring to autumn [42].
*Predator*	“Mainly dolphins (atuninha or toninha), “sharks, whales, conger eel (safio) and yellowfin tuna (albacora)”; “yellow-legged gull (gaivota)”, albatross and other birds”.	Common dolphin (*D. delphis*) [59–62]; species of demersal fish, seabirds and marine mammals [41, 42, 59, 63, 64].
*Prey*	“Plankton, algae called “limo”, small shrimp, krill, the spawn of other fish species and their own spawn”.	Zooplankton as their energy source [58, 68]; Phytoplankton [58, 69]; fish eggs and crustaceans [58]. Sardines may predate on their own eggs in winter [69].

European pilchard show migratory behaviour, a high dispersal capacity and schooling behaviour similar to other pelagic fish [45]. In the present study, respondents exclusively reported this type of behaviour pattern. There was an account of a fisherman who referred to this ability to school as a way to ward off predators. Neilson and Perry (1990) show that the presence of competitors or predators may change the direction or influence the intensity of these migrations in schooling [46] (Table 5).

In Portugal, the geographic distribution of this pelagic fish covers the entire coastline, Madeira Island and the Azores [47]. However, sardine migration patterns are not yet well understood [42]. There are indications of seasonal migrations along the Portuguese Coast [48], and fifteen interviewees mentioned this information.

According to acoustic campaigns performed in April and May of 2015 by the Portuguese Sea and Atmosphere Institute (IPMA), the abundance of sardines decreased from the north to the south of Portugal [28]. Regarding sardine migrations, it is known that they occur during the growth phase and towards the north coast of Spain [23]. During the collection of ethnoecological data in this part of the interview, it was observed that there was a variation in the responses among the interviewees. There were 18 fishermen who said that the sardines come from the north of Portugal and another 32 fishermen who say that they come from the north and the south of Portugal. This pattern of responses among fishermen reinforces the need to investigate and explore the studies on the migratory behaviour of sardines in the Iberian Peninsula.

The European pilchard show migratory behaviour, a high dispersal capacity and schooling behaviour similar to other pelagic fish is distributed mainly near Póvoa de Varzim and Figueira da Foz in the northwestern region of Portugal and near Peniche and Lisbon in the southwestern region [49]. In the Algarve (Southern Portion of Portugal), this pelagic fish is found in greater quantities in Lagos, Portimão and between Faro and Vila Real de Santo António [49]. According to the fishermen of Peniche’s LEK, the Figueira da Foz region and the Algarve were cited the most frequently when asked about where the sardine is on the coast after passing through Peniche (Table 5). The regions of Portimão, São Pedro de Manoel, Olhão, Tocha Beach and Sagres were identified by the fishermen in the southern portion of Portugal, which are included in or near the range of greater distribution found during the last acoustic campaign by the IPMA. None of the respondents specifically mentioned V. Real de Santo Antônio.

### Development of sardines

The sardines show a very fast growth rate [42, 45, 50], growing to approximately 90% of their full size in 2 years [42]. Most fishermen in the community of Peniche (*N* = 57) corroborate the scientific research in relation to rapid growth (Table 5). In relation to the stages of development of this species, the fishermen only mentioned the egg phase as constituting the whole sardine life history. However, it is known that the larval stage is one of the development stages of this pelagic fish [51].

The European pilchard matures early [52, 53]. Individuals are largely mature at 1 year of age, and all individuals are reproductively mature at 2 years of age [41, 52]. In the present study, only 19 of fishermen said that sardine reach sexual maturity at 1 year of age and 44 of respondents mentioned that they are ready to reproduce from 3 to 7 months of age (Table 5).

### Spawning and fat accumulation season

Regarding reproduction, spawning of sardines occurs in batches of eggs [54, 55]. The spawning of the Atlantic sardines occurs mainly on the western coast of Portugal between the Nazaré Canyon and the Minho river and in the Cantabrian Sea [56]. Along the western Iberian coast, the spawning season of this pelagic species ranges from September to May, with spawning peaking in November to the north of Portugal from October to April [56]. Nunes and collaborators (2011) showed that the spawning peak occurs mainly between December and February along the Portuguese coast. According to fishermen from Peniche, the main months of spawning are also December, January and February. This period as a whole (from October to April) included the months most cited by the respondents (Table 5).

There is a variation in the duration of spawning times in the European waters of the North-East Atlantic, ranging from 3 months per year up to 8 months to the south and west of the Iberian Peninsula for large fish [43]. A similar pattern was found in responses of our interviewees from Peniche, in which 72 fishermen said that the Atlantic sardine spawn time can range from one to 8 months (Table 5).

The winter was cited by 12 fishermen as being the season during which sardine spawn the most along the Portuguese Coast, and others (*N* = 12) said that the spawning occurs 2 to 4 times a year. This point aligns with the scientific information that Atlantic sardines exhibit a prolonged spawning period during the year, with more pronounced spawning mainly in the colder months of the year [43] (Table 5).

*S. pilchardus* begins to store fat reserves before the breeding season between late summer and autumn [58]. However, sardines can also accumulate fat from late spring to autumn [42]. Most months cited by fishermen (June through October) fit the range of months for the accumulation of fat found in the scientific literature (Table 5).

### Trophic ecology: Predator and prey

The sardine is one of the main prey species of the common dolphin (*D. delphis*) [59–62]. It also serves as a food base for several species of demersal fish, seabirds and marine mammals [41, 42, 59, 63, 64]. These animals were also cited by fishermen during the interviews (Table 5). The common dolphin stood out among all the other predators [59–62]. Along the Portuguese Coast specifically, *S. pilchardus* was the most important species in the common dolphin diet in a study in which the stomach contents of this animal were examined during accidental catch and when they were stranded [62]. It is also known that the common dolphin is an opportunistic predator of small epipelagic fish [61], usually in places with moderate or high productivity [65] where sardines seek their energy sources [44]. Given the current population decline in the sardine population on the Iberian coast [66], attention should be given to the conservation of the common dolphin along the Portuguese coast, as this is one of the main predators of this clupeoid fish [67].

Sardines primarily seek zooplankton as their energy source [58, 68]. Phytoplankton are also an integral part of the diet of this species [58, 69]. Among the zooplankton, we should highlight copepods, decapods and cirripeds [58, 69], fish eggs and crustaceans [58]. During the winter spawning months, sardines may exhibit cannibalistic behaviour in which they predate on their own eggs [69]. Respondents describe a foraging behaviour similar to that reported in the investigative work on European sardine prey (Table 5). In general, it is also observed that fishermen in the fishing village of Peniche show sardines with migratory behaviour, high dispersal capacity, prey and school behaviour similar to other pelagic fish.

### Human uses, beliefs and food taboos about European pilchard

The great majority of fishermen (*N* = 81) in our ethnoecological study indicated the great economic importance of Iberian sardines, *S. pilchardus,* to the local community of Peniche. According to data from the Statistics Yearbook of the Central Region of 2015 made by Portugal’s National Statistical Institute (INE), sardine fisheries officially yielded approximately 1223 tons of fish and 3517 thousand Euros for the municipality of Peniche in 2015 [7]. This fact proves the strength of this economic activity for this region.

The summer was highlighted among the respondents as being the most important season for sardine fishing, local commerce and tourism. In this season, the sardines are in the fattening stage [42, 58], and their fat content is high [70]. The sardine in this period has a flavour and aroma that is more appreciated by the consumers [71], which makes this species more economically profitable. When the fat content is low (2–5%), this fish is less preferred by consumers and is normally sent to the canning and fish meal industries [70]. In other seasons, sardines are primarily used as baitfish for demersal fishing or for the canning industry [72]. All sardine uses known to the scientific literature were also mentioned by the fishermen during the interviews.

Taboos are unwritten social rules that regulate human behaviour and can both govern and affect human social life and serve to manage a local biological resource [73]. In the local community of Peniche, there were no significant taboos or food aversions regarding sardines by fishermen (*N* = 6). In local communities of the Amazon and in the Atlantic Forest in Brazil, taboos and aversions were also not associated with herbivorous fish or invertebrate eaters [74]. According to Begossi (2004), this can be an adaptive strategy of local inhabitants to fish of higher trophic levels that can more easily accumulate toxins by eating a variety of prey (plants, invertebrates and other fish) [74]. This may be one of the reasons that there are few fishermen with taboos or sardine aversions. Another hypothesis is that these social rules may be losing strength over the years due to the exodus of fishermen to other economic activities, many of whom leave due to the low economic profitability of this profession and the difficulties imposed by European Union legislation on Portuguese artisanal fisheries [75].

Regarding medicinal purposes, 9 fishermen indicated that sardine can treat high cholesterol levels and patients with cardiac problems. In a study about the fauna and the role of taboos in the conservation of animals in a forest reserve in southeastern Cameroon [76], sardine (*Sardina sp.*) are used medicinally to treat cardiovascular diseases, which was also indicated in our survey by some fishermen (*N* = 9). In the Sierra de Segura (Albacete, Spain), *S. pilchardus* is used for medicinal purposes in the treatment of sore feet and blisters in modern times and is marketed in the Spanish markets as food [77].

### Conservation concerns and co-management

There is a growing interest in LEK research in order to provide complementary data for several small-scale fishery species [78]. With the difficulties and vulnerability of the marine ecosystem, LEK correctly acquired and aggregated at appropriate spatial-temporal scales becomes an important marine species conservation tool [79]. The management of these coastal resources in fishing villages can also be better achieved by exploring data of this nature, which provide information on the ecology, behaviour and presence of these species in the environment [80].

In the present work, we present LEK data on the ecology of sardines that corroborated scientific research. Other data generated in the interviews that were not validated by the scientific literature can be tested and incorporated into new hypotheses before carrying out a scientific study. According to Drew (2005), an analysis of the components of traditional ecological knowledge can reveal new information and thus contribute to the formulation of testable hypotheses in order to improve scientific infrastructure. Silva et al. (2014) also reveal the importance of generating new scientific questions through ethnoecological data [81]. The LEK about sardines acquired here may complement pre-existing scientific data. Due to the high cost and lack of resources for investments in traditional samplings, data of this nature become important for conservation practices and development [82].

Given the low stock levels of the sardine population in the Iberian Region [66], participation among the actors involved in fishing regulations should be carried out in an interactive manner [83]. Ecological knowledge data on spawning and the time of fat accumulation may provide researchers an additional source of data to better understand the reproductive behaviour of this species from the fishermen’s point of view. Analysing this knowledge can contribute to a better understanding and reduction of internal conflicts between fishing managers, politicians and the community about the correct time for harvesting this species. In addition, LEK data acquired from these traditional communities are important because it reveals the most recent changes in environmental processes [84].

The new data (trophic ecology, habitat and behaviour) that emerged during the interviews can serve as a starting point for research on the population structure of sardines. On a larger spatial scale, this kind of data, with adequate treatment, becomes important in the construction of management and conservation plans for sardines. Finally, we can note that the LEK of the sardines in Peniche should (and did) treat all stakeholders as being within a continuously adaptive framework [85].

## Conclusions

The socioeconomic profile of the fishermen of Peniche was described in this ethnoecological research. Respondents provided detailed informal data on the taxonomy, ecology and biology of *Sardina pilchardus*. This informal knowledge showed agreements with the scientific literature. We suggest the use of non-corresponding data with formal knowledge to aid in the construction of testable hypotheses for new investigative work on sardines. The data generated here can be used to try to improve the understanding of the fishermen’s knowledge of the European pilchard by managers and conservationists. This approach requires the use of an adaptive framework, which contributes to an improvement in the relations between the actors involved with the resource. The food taboos and social rules about European pilchard were not relevant to conservation in this community. This species, which is mainly used as baitfish for other fishes and in the canning and fish meal industries, provides a great economic value for the fishing community studied here.

Finally, our results highlighted that artisanal fishermen from Peniche show ethnoecological data about European pilchard that can support scientific knowledge, as well as collaborate with future initiatives in pursuit of viable conservation goals for sardines on the coast of Portugal.

## Additional files

Additional file 1: Statement of Informed Consent (IC) and agreement to participate in the research. (DOCX 16 kb)

Additional file 2: Script of interview. (DOCX 14 kb)

## Abbreviations

FAO: Food and Agriculture Organization of the United Nations
IC: Statement of Informed Consent
INE: Portugal’s National Statistical Institute
IPMA: Portuguese Sea and Atmosphere Institute
IUCN: International Union for Conservation of Nature
LEK: local ecological knowledge
MNR: Berlengas Marine Natural Reserve
