# Supplementary material for: Sharing fishers´ ethnoecological knowledge of the European pilchard (Sardina pilchardus) in the westernmost fishing community in Europe
Source: J Ethnobiol Ethnomed. 2017 Sep 14;13:52. doi: 10.1186/s13002-017-0181-8 (PMC5599890; doi:10.1186/s13002-017-0181-8)
Supplement: Supplementary file 1 — Statement of Informed Consent (IC) and agreement to participate in the research. (DOCX 16 kb) [file 13002_2017_181_MOESM1_ESM.docx]

## **Additional file 1:** Statement of Informed Consent (IC) and agreement to participate in the research.

**TERMO DE CONSENTIMENTO LIVRE E ESCLARECIDO**

Caro Senhor,

Meu nome é Heitor de Oliveira Braga. Sou estudante da Universidade de Coimbra e estou a fazer um estudo sobre o conhecimento da sardinha com pescadores aqui em Peniche. Tal pesquisa pretende saber as características da pesca artesanal, assim como buscar o conhecimento ecológico local dos pescadores sobre as áreas em que as sardinhas aparecem mais, os locais onde elas provavelmente se reproduzem entre outros aspectos do seu ciclo de vida. Também irei pesquisar as atitudes dos pescadores em relação a conservação desta espécie.

Para a realização da pesquisa preciso conversar com alguns pescadores em Peniche como o senhor. Se sentir-se à vontade para colaborar com nossa pesquisa irei aplicar um questionário com perguntas relacionadas a sardinha**.** Se o senhor permitir, as entrevistas poderão ser gravadas por meio de um aparelho eletrônico. Essas gravações com as informações registradas poderão ser repassadas para um cd e guardadas em meu local de trabalho na Universidade. Caso seja autorizado, irei tirar algumas fotos da arte de pesca, da ação de pesca e da espécie de animal estudada em alguma eventualidade. Essas imagens somente poderão ser divulgadas em revistas ou reuniões científicas como imagens ilustrativas. Se no meio da entrevista o senhor desistir de colaborar com o nosso estudo não será prejudicado de forma alguma. A sua identidade será mantida em sigilo e apenas as informações serão registradas. Se o senhor permitir, seu nome será coletado e anotado nos roteiros de entrevistas. Sendo necessário entrevistar o senhor novamente para coletar outras informações, esses dados pessoais irão facilitar meu o estudo.

A sua opinião será muito importante para o nosso estudo, pois os dados coletados nas entrevistas poderão contribuir para a conservação do meio ambiente, para o conhecimento ecológico local sobre as sardinhas e para que a cultura e a tradição dos pescadores artesanais não se percam ao longo das gerações. As informações coletadas irão fazer parte de um trabalho que poderá ser publicado em revistas científicas, além de constituir um relatório para ser entregue na Capes Brasil. Posteriormente essas informações serão divulgadas aos pescadores de Peniche para o conhecimento de todos, através de banners ou folhetos auto explicativos. Caso o senhor concorde em participar da entrevista, peço que o senhor assine este termo de consentimento, que também será assinado por mim, que sou o pesquisador responsável. Dessa forma, uma cópia ficará comigo e a outra com o senhor. Caso o senhor não queira assinar, sua decisão será respeitada. Em caso de dúvida, por favor pergunte. Desde já agradeço. Meu endereço de trabalho é na Universidade de Coimbra, Departamento da Ciências da Vida, Centro de Ecologia Funcional, Coimbra, Portugal, CEP: 3000-456, Telefone: +351918562333 e Fax: 239 855211. E-mail: [heitorob@gmail.com](mailto:heitorob@gmail.com).

Eu, __________________________________, idade: ____, aceito participar da pesquisa intitulada **“Conhecimento ecológico local (CEL) de pescadores em áreas marinhas protegidas (AMP´s) no Oceano Atlântico (Brasil e Portugal): Sardinha-verdadeira e Sardinha Européia”,** tendo sido devidamente informado e esclarecido, como disposto acima.

__________________________________________ _______________________________________

Heitor de Oliveira Braga (Pesquisador Responsável) Assinatura do voluntário

______________________, ____ / ____ / ___­_.

Local dia mês ano

A rogo do Sr(a)_________________________________, assinam: “marca do polegar”

___________________________ ____________________________

Assinatura da Testemunha 1 Assinatura da Testemunha 2
