# Supplementary material for: Sharing fishers´ ethnoecological knowledge of the European pilchard (Sardina pilchardus) in the westernmost fishing community in Europe
Source: J Ethnobiol Ethnomed. 2017 Sep 14;13:52. doi: 10.1186/s13002-017-0181-8 (PMC5599890; doi:10.1186/s13002-017-0181-8)
Supplement: Supplementary file 2 — Script of interview. (DOCX 14 kb) [file 13002_2017_181_MOESM2_ESM.docx]

**Additional file 2:** Script of interview

*****Before the interview is carried out the reading of Statement of Informed Consent (IC).

Date: ____/____/____. Number of interview: _____.

Part I. Profile of fishermen

1- Name and/or Nickname (Optional):

2- Location of the interview:

3- Age:

4- Level of education: ( ) illiterate; ( ) A (1st Cycle: 1-4 years of study); ( ) B (2nd Cycle: 5-6 years of study);

( ) C (3rd Cycle: 7-9 years of study); ( ) D Secondary Education: 10-12 years of study; ( ) Higher education

5- Time of residence in Peniche?

6- Fishing currently?

7- Fishing time?

8- Income source? Other source of income?

9- Do you have a boat?

10- Type and length of boat you use for fishing?

11- Fishing time at sea?

12- Other relevant information on fisheries?

13- Sardine fishing schedule?

Part II: Local ecological knowledge (LEK) about the European sardine (Sardina pilchardus) in Peniche, Portugal.

1- Folk taxonomy or popular taxonomy?

2- Preferential Habitat (Where does the sardine live?)

3- Route and Migration (Where does the sardine go and where does it come from? Ideal route?)

4- Depth Interval (m) - (How deep is it?)

5- Behavior (Form schools?)

6- How long (months or years) are the sardines already ready to reproduce?

7- Time of spawning / breeding (months or season). Discuss spawning of Sardine.

8- Development - How do you grow (slow, fast, etc.)? At some point in your life are larvae, eggs?

9- When (months or season) they accumulate fat (they grow and fatten)?

10- Do they have a long or short life?

11- Predators (Who eats it?)

12- Preys (What does sardine eat?)

13- Economic and personal importance (low, medium or high)?

14- What are the uses of sardines? (Bait, market, no utility, etc.)

15- What time of year (months) would you choose to stop sardine fishing?

16- For medicinal purposes? Craft purposes? Food restriction? Medicine purpose?
